# Penetrating Endplate Screw Fixation for Thoracolumbar Pathological Fracture of Diffuse Idiopathic Skeletal Hyperostosis

**DOI:** 10.1155/2022/5584397

**Published:** 2022-02-24

**Authors:** Tetsuhiro Ishikawa, Mitsutoshi Ota, Tomotaka Umimura, Takahisa Hishiya, Joe Katsuragi, Yasuhito Sasaki, Seiji Ohtori

**Affiliations:** ^1^Orthopaedic Surgery, Sanmu Medical Center, Chiba, Japan; ^2^Orthopedic Surgery, Seirei Yokohama Hospital, Kanagawa, Japan; ^3^Department of Orthopedic Surgery, Chiba University, Chiba, Japan

## Abstract

With the advancement of cancer treatment and minimally invasive surgery, the indications for surgery for metastatic spinal tumors are expanding. Diffuse idiopathic skeletal hyperostosis (DISH) is a noninflammatory skeletal disease characterized by calcification and ossification of ligaments and entheses. In Japan, the prevalence of DISH is increasing with its superaging society. The purpose of this article is to report a case of applying a novel screw technique for pathological fracture in a patient with DISH and spinal metastasis. An 80-year-old man with spinal metastasis presented with acute onset of severe back pain, and investigations revealed a fracture of a metastatic lesion in T10–T12 in the range of DISH. We performed posterior fixation with a percutaneous pedicle screw system using a penetrating endplate technique. The patient's back pain improved, and he was able to mobilize with minimal assistance and survived for 8 months with a good quality of life. Spinal fracture accompanied by DISH sometimes occurs with severe instability because of injury across 3-column injury and its long lever arm. Spinal instability neoplastic score indicates instability of pathological fractures of spinal metastases but needs to be evaluated carefully when DISH is present. The prevalence of DISH is increasing in the elderly, and penetrating endplate screws can be an effective option in posterior fusion surgery for patients with DISH and spinal metastases.

## 1. Introduction

Surgical treatment of spinal metastases is largely palliative. The aim of palliative surgery in such cases is to improve pain control and reduce or eliminate neurological deficits, thus improving the patient's quality of life [1]. With the advancement of cancer treatment and minimally invasive surgery, the indications for surgery for metastatic spinal tumors are expanding [2]. As a result, spine surgery is increasingly being performed for spinal metastasis in older patients. However, for spine surgery in the elderly, there is a discrepancy between less-invasive surgery and the acquisition of fixation strength, which can cause difficulties in deciding surgical methods.

Diffuse idiopathic skeletal hyperostosis (DISH) is a noninflammatory skeletal disease characterized by calcification and ossification of ligaments and entheses [3]. Resnick et al. [4] defined DISH as ossification of the spine across at least 3 disc spaces without disc degeneration, with relative preservation of disc height in the vertebral segments involved. In Japan, which is experiencing a superaging society, the prevalence of DISH is increasing [5–9]. Hiyama et al. reported that the prevalence of DISH in subjects aged ≥70 years was 40.9% [8].

Recently, several studies reported the efficacy of a transdiscal screw technique with its strong fixing force for some instances of lumbar degenerative disease [10–15]. DISH-related fractures are a suitable indication for transdiscal screws because bony fused segments do not have flexibility or disc function.

We have applied a transdiscal “penetrating endplate screw (PES) technique” to fix thoracolumbar DISH-related fractures in patients with spinal metastasis.

## 2. Case Report

**Figure 1:**
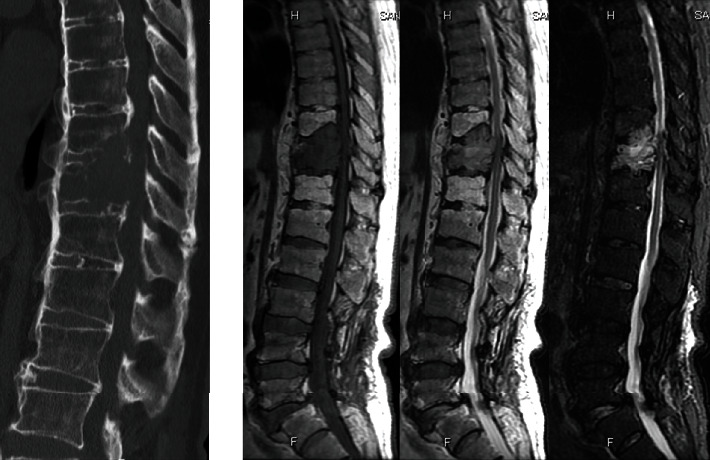
An 80-year-old man with renal cell carcinoma (RCC) and metastasis to the thoracic spine, lungs, and lymph nodes was paralyzed in both lower limbs 5 years earlier.

**Figure 2:**
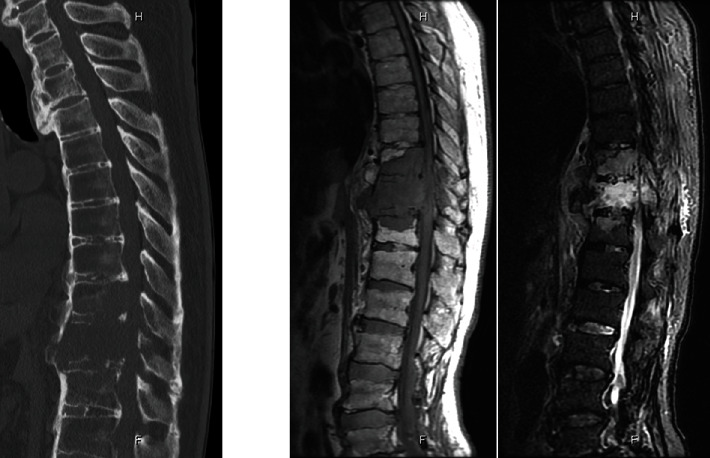
He presented with acute onset of severe back pain. CT and MRI reveal a fracture of the metastatic lesion in T10–T12.

**Figure 3:**
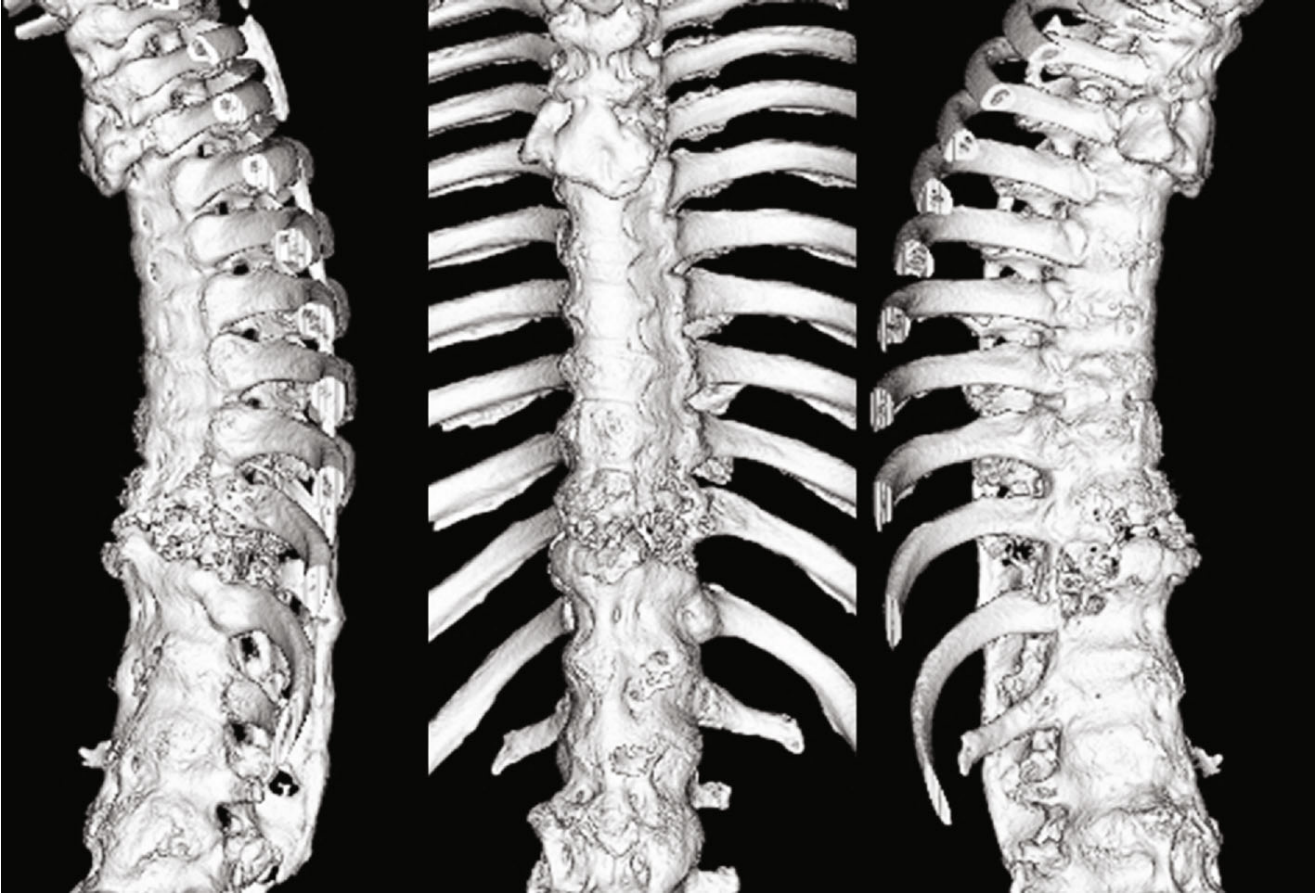
3D CT showing the pathological fracture occurred within the range of DISH, which suggested a hyperinstability.

**Figure 4:**
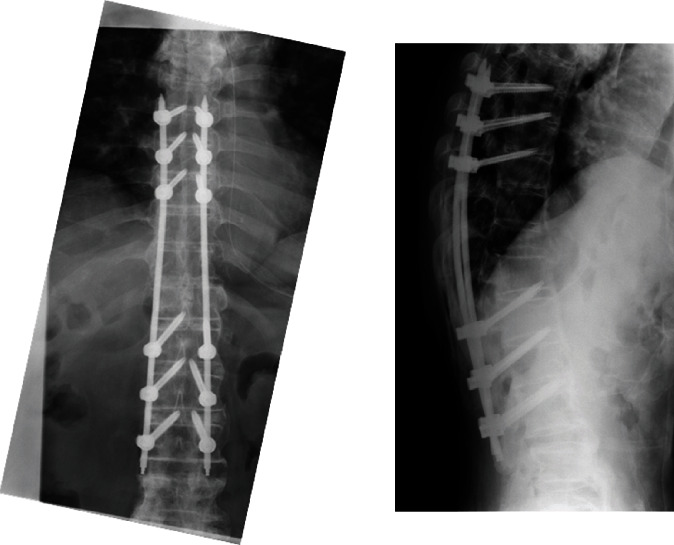
Percutaneous pedicle screw stabilization using penetrating endplate screw technique was performed 3 levels above and below the affected vertebrae.

**Figure 5:**
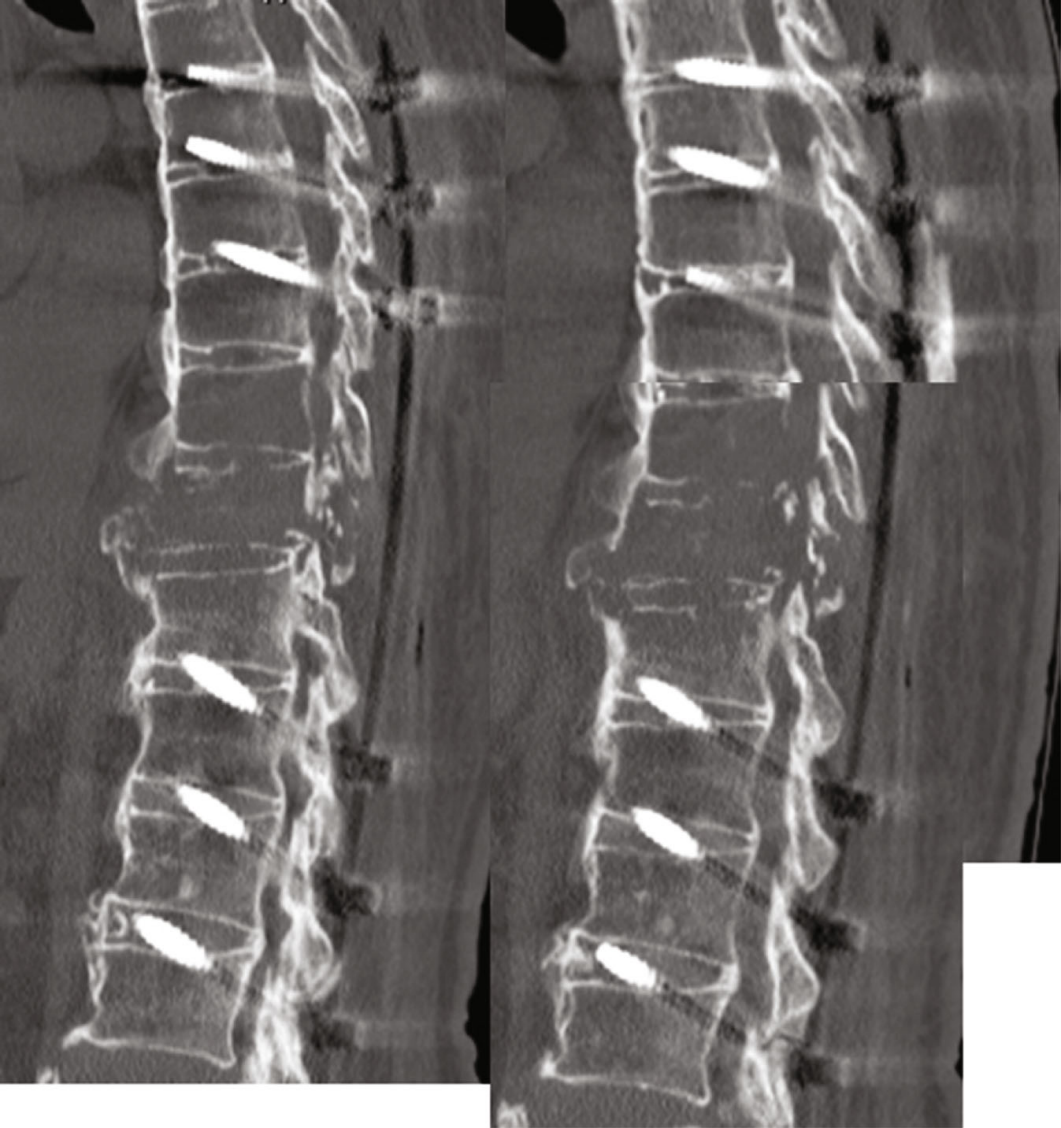
Postoperative CT showing appropriate implant placement and satisfactorily strong stabilization.

An 80-year-old man had a history of cervical OPLL surgery and multiple lumbar degenerative disease surgeries. He had been diagnosed with renal cell carcinoma (RCC) and underwent surgery to excise the tumor 12 years ago. He received radiation therapy and bone-modifying drug therapy 5 years later for thoracic spine metastasis, lung metastasis, and lymph node metastasis (Figure 1). The patient was paralyzed in both lower limbs (Frankel B) and had been living with repeated respite admissions to a palliative ward for 2 years. One day, he presented with acute onset of severe back pain and investigations revealed a fracture of a metastatic lesion in T10–T12 (Figure 2). A 3D CT scan shows the pathological fracture occurred within the range of DISH, which suggested a hyperinstability of the spine (Figure 3). The patient was evaluated according to the spinal instability neoplastic score (SINS) modified Tokuhashi score and modified Katagiri score (Table 1) [16, 17]. According to the oncology team, his likely survival was more than 6 months. At first, the patient refused the surgery when our team recommend posterior fixation surgery; however, he could not move or even sit up because of severe pain. The VAS pain score and ODI score were 95 and 91.1, preoperatively. So ultimately, surgery was planned at the request of both the patient and his family. Percutaneous pedicle screw stabilization using a penetrating endplate screw technique was performed 3 levels above and below the affected vertebra (Figure 4). Postoperative CT showed proper implant placement and satisfactory strong stabilization (Figure 5). The patient's back pain improved dramatically, and he was able to mobilize with minimal assistance. The postoperative scorings improved that VAS was 30 and ODI was 68.9 at 1 month after surgery. He survived for 8 months with a relatively good quality of life, without severe pain.

## 3. Discussion

Here, we have applied a transdiscal screw to fix a thoracolumbar DISH-related fracture in a patient with spinal metastasis. In recent years, rapid progress has been made in the treatment of malignant tumors, and with the development of anticancer drugs, widespread use of tumor-targeted therapy, and the advent of molecular-targeted drugs, patients with cancers who were previously expected to have a prognosis of only a few months once metastasis to the spine was confirmed can now be expected to survive for years [1, 2]. Therefore, the usefulness of palliative surgery has been reconfirmed due to the prolonged prognosis and minimally invasive surgery in cases where the prognosis was previously not >6 months and paralysis was not an indication for surgery in the past. Moreover, in recent years, minimally invasive surgery has become more widespread in the field of spine surgery with the development of various surgical techniques, such as the use of percutaneous pedicle screws (PPS), minimally invasive transforaminal lumbar interbody fusion (MIS-TLIF), and lateral lumbar interbody fusion (LLIF) [18–20].

Patients with DISH-related vertebral fractures are at risk of developing delayed paralysis due to their immobilized spinal column and significant fracture site instability with long lever arms [21–24]. Surgical treatment providing rigid fixation is recommended, and several studies have shown that posterior spinal fixation, extending for a minimum of 3 levels above and below the injury, results in reliable fracture healing [21–24]. However, an ideal surgical procedure for vertebral fracture in patients with DISH remains to be established.

Additionally, patients with DISH tend to be of advanced age with multiple comorbidities, indicating a high rate of complications and perioperative mortality [21–23]. Rigid fixation and minimally invasive surgical procedure are attractive when treating DISH-related spinal fractures. Therefore, we have applied a transdiscal penetrating endplate screw (PES) technique to fix thoracolumbar DISH-related fractures in patients since 2015. A retrospective study was conducted to validate the effectiveness of posterior fixation using the PES technique for thoracolumbar DISH-related fractures [25].

In this case, the patient's SINS was 12: intermediate instability. However, the patient had DISH in his thoracolumbar spine, so the instability was considered to have been higher than indicated by the SINS. Spinal fracture accompanied by DISH sometimes occurs with severe instability because of injury across 3 columns and its long lever arm. In general, SINS indicates instability of pathological fractures of spinal metastases. However, we need to be careful to evaluate the patients with fractures when DISH is present in the patient's spine because its instability is higher than fracture patients without DISH.

In summary, we experienced thoracolumbar pathological fracture in an elderly patient. Minimally invasive and strong fixation was thought to be desirable. We applied penetrating endplate screws using a percutaneous system and were able to eliminate back pain effectively, using minimally invasive surgery.

## Figures and Tables

**Table 1:** Patients' clinical summary and evaluation based on scoring system.

Sex	Male
Age	80 years
Paralysis	Frankel B
Modified Katagiri score	7
Modified Tokuhashi score	5
SINS	12

## Data Availability

The paper describes a case report of a patient that was treated by the authors.
